# Association and mediating mechanism between remnant cholesterol and first-ever stroke among the Chinese general population

**DOI:** 10.3389/fnins.2023.1161367

**Published:** 2023-05-25

**Authors:** Heng Li, Shuai Miao, Lu Chen, Bin Liu, Yan-Bin Li, Rui-Sheng Duan

**Affiliations:** ^1^Department of Neurology, Shandong Provincial Qianfoshan Hospital, The First Affiliated Hospital of Shandong First Medical University, Jinan, China; ^2^Medical School of Chinese People’s Liberation Army (PLA), Beijing, China; ^3^Department of Neurology, The First Medical Center, Chinese PLA General Hospital, Beijing, China; ^4^Department of Neurology, ZiBo Central Hospital, Zibo, China; ^5^Shandong Institute of Neuroimmunology, Jinan, China

**Keywords:** stroke, remnant cholesterol, hypertension, propensity score methods, mediation analysis

## Abstract

**Background:**

Remnant cholesterol (RC) has been suggested to be implicated in atherosclerosis. The objective of the study was to evaluate the association between RC and first-ever stroke in the Chinese general population and to investigate whether the association is mediated *via* hypertension or diabetes.

**Methods:**

This study is a retrospective cohort analysis of participants from the China Health and Nutrition Survey. Participants without previous stroke and myocardial infarction in 2009 were enrolled and followed up in 2011 and 2015. Logistic regression analyses were adopted to explore the association of RC with stroke risk. Propensity score methods and doubly robust estimation method were used to ensure the robustness of our findings. Potential mediators were identified by mediation analyses.

**Results:**

A total of 7,035 participants were involved, and during 6 years of follow-up, 78 (1.1%) participants experienced a first-ever stroke. Participants with high RC had a significantly higher incidence of stroke (1.4% versus 0.8%; *p* = 0.007). High RC was associated with 74% higher stroke risk after adjusting for multiple relevant variables (odds ratio [OR], 1.74; 95% CI, 1.06–2.85). The association was consistent in analyses using propensity score methods and doubly robust estimation method. Hypertension showed a significant mediating effect on the association between RC and stroke, while the mediating effect of diabetes was not significant.

**Conclusion:**

High RC increased the risk of first-ever stroke in the Chinese general population without previous stroke and myocardial infarction, partially through the pathway of hypertension. RC might be a potential target for the primary prevention of stroke.

## Introduction

Stroke remained the second-leading cause of death and the third-leading cause of death and disability combined worldwide in 2019. Despite substantial reductions in age-standardized rates from 1990 to 2019, the burden of stroke remains high worldwide (Feigin et al., 2021). According to a nationwide survey conducted in 2020, the estimated prevalence, incidence, and mortality rate for stroke among the Chinese population aged 40 years old and older were 2.6%, 505.2 per 100,000 person-years, and 343.4 per 100,000 person-years, respectively, indicating an ongoing challenge in China (Tu et al., 2023). Medical therapies, especially strategies targeting low-density lipoprotein (LDL), have demonstrated efficacy in the prevention of cardiovascular events; however, substantial residual risks still exist (Shaya et al., 2022). Other lipid-related factors beyond LDL, such as triglyceride (TG) and low high-density lipoprotein (HDL), have fostered an interest in hopes of seeking important contributors of the residual risk. Epidemiological data support associations of TG and low HDL with the development of cardiovascular diseases, and it was hypothesized that substantially increasing HDL or decreasing TG would lead to risk reductions (Tall et al., 2022). However, the causal role was debated and clinical trials have shown discouraging results (Tall, 2021; Liang et al., 2022).

Recently, the atherogenic roles of triglyceride-rich lipoprotein (TRL) and remnant cholesterol (RC) have attracted increased attention. RC is the cholesterol content of TRL, composed of chylomicron remnant, very-low-density lipoprotein, and intermediate-density lipoprotein (Varbo and Nordestgaard, 2017). It accumulates in the subendothelial space after penetrating the artery wall and leads to a variety of vascular damage, including endothelial dysfunction, inflammation, and ultimately atherosclerosis (Varbo and Nordestgaard, 2017; Sandesara et al., 2019).

Emerging evidence has identified RC as an independent risk factor for cardiovascular diseases (Nordestgaard, 2016; Castañer et al., 2020; Langsted et al., 2020; Quispe et al., 2021). However, only a few studies describe the association between RC and stroke (Varbo and Nordestgaard, 2019; Langsted et al., 2020; Chen et al., 2021; Li et al., 2022). As risk factors of coronary heart disease and stroke differ in the degree or direction of associations (Matsunaga et al., 2017), more evidence is needed to clarify the effect of RC on stroke. The present nationwide population-based cohort study aimed to elucidate the association of RC with first-ever stroke in the general Chinese population. We also explored whether hypertension or diabetes mediated the association between RC and stroke. This study may provide supportive evidence for the association of RC with stroke and elucidate potential underlying mechanisms.

## Methods

### Study design and participants

Data of the present study were derived from the China Health and Nutrition Survey (CHNS). The CHNS is an ongoing nationwide longitudinal open cohort study established in 1989, with the aim of capturing sociological, economic, and demographic factors that influence health and nutritional status across the Chinese lifespan (Popkin et al., 2010). Follow-up surveys on the same population were conducted every 2–4 years. A total of 11 waves (1989, 1991, 1993, 1997, 2000, 2004, 2006, 2009, 2011, 2015, and 2018) have been conducted thus far. In each wave, demographic, socioeconomic, lifestyle, nutritional and health information were collected. During the three waves of investigation in 2009, 2015, and 2018, blood samples were added at the same time.

**Figure 1:**
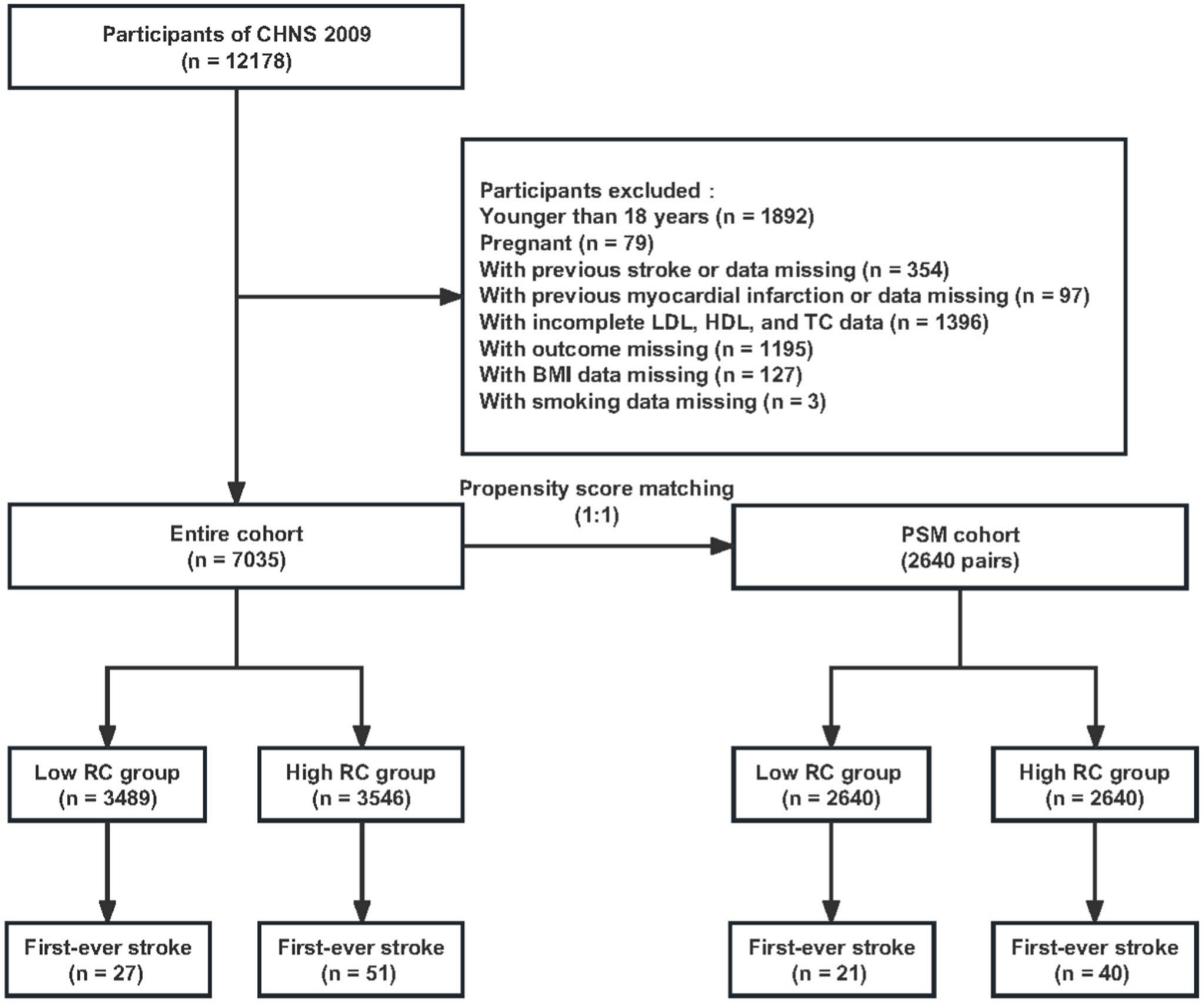
Flowchart of participant selection. CHNS, China Health and Nutrition Survey; TC, total cholesterol; HDL, high-density lipoprotein; LDL, low-density lipoprotein; BMI, body mass index.

Our analyses used survey data from three waves (2009, 2011, and 2015) of the CHNS, because follow-up data in 2018 was not available at the time of our analysis. Data derived from the 2009 wave was analyzed as baseline and the participants were followed in the subsequent waves of 2011 and 2015. At the 2009 wave of the survey, 12,178 participants who finished both the questionnaires and physical examination were included. We excluded those under 18 years old (*n* = 1892) or pregnant (*n* = 79), with previous stroke or without stroke data (*n* = 354) and with previous myocardial infarction or without myocardial infarction data (*n* = 97). Participants with incomplete LDL, HDL, and total cholesterol (TC) data (*n* = 1,396) and participants whose outcome (first-ever stroke) cannot be confirmed during the follow-up (*n* = 1,195) were further excluded. Of these participants, 130 individuals with missing covariates [body mass index (BMI), *n* = 127; smoking, *n* = 3] were excluded. Finally, a total of 7,035 individuals were included in the entire cohort. The flowchart of the screening process is shown in Figure 1. All participants enrolled in the study signed written informed consent before they participated in the survey.

### Data collection and measurement

Demographic characteristics (age and sex), medical history (hypertension, diabetes, history of stroke or myocardial infarction), smoking status, drinking status and current medication were self-reported on a questionnaire at baseline. Blood pressure was measured three times and the mean was used in the final analysis. Height and weight were measured while the participants were wearing light clothing and standing without shoes. BMI (kg/m^2^) was calculated as body weight (kg) divided by the square of height (m^2^).

Venous blood samples were collected from fasting participants and the levels of TC, HDL, LDL, high-sensitivity C-reactive protein (hs-CRP), glucose, and glycated hemoglobin A1c (HbA1c) were measured.

### Definition of exposure, covariates and outcome

There is no standard method to estimate RC until now, and calculated RC has been frequently used in previous studies because it can be obtained from the standard lipid profile at no additional cost (Nordestgaard et al., 2007; Joshi et al., 2016; Elshazly et al., 2020). In the present study, RC was calculated as TC minus LDL and HDL. Hypertension was defined as exhibiting a self-reported history of hypertension or receiving antihypertensive medication or mean blood pressure ≥ 140/90 mmHg in the physical examination. Diabetes was defined if the participant meets at least one of the following: (1) a previous diabetes history; (2) receiving hypoglycemic medication (oral agents or insulin injection); (3) a fasting blood glucose ≥ 7.0 mmol/L; (4) an HbA1c ≥ 6.5%. The outcome of the present study was the first-ever stroke that occurred during the follow-up. Stroke was defined based on a self-report of diagnosis by the question “Has a doctor ever given you the diagnosis of stroke?” In the analysis, the first-ever stroke was confirmed when a participant included without a stroke history in the 2009 wave reported a new history of stroke during the follow-up conducted in 2011 and 2015.

### Statistical analysis

The full set of original participants comprising 7,035 individuals constituted the entire cohort. Individuals were divided into two groups according to the median of RC. A propensity score matching (PSM) cohort was used to minimize the potential confounding effect. Covariates identified as plausible confounders of stroke were included in the propensity score models (Williamson and Forbes, 2014; Buajitti and Rosella, 2022). The propensity score based on age, sex, smoking, drinking, diabetes, hypertension, BMI, LDL and hs-CRP was developed using the multivariable logistic regression model. Participants with high RC were matched 1:1 to those with low RC according to the propensity score using the nearest neighbor matching algorithm with a caliper width of 0.2. A total of 2,640 pairs were included in the PSM cohort. The standardized mean differences (SMD) were calculated to evaluate the baseline differences between the high RC group and the low RC group, where a value <0.1 was considered balanced after the matching. The *z*-test was used to evaluate the difference in stroke incidence between the high RC group and the low RC group.

Logistic regression was applied in the entire cohort and PSM cohort, and odds ratio (OR) with 95% confidence intervals (CI) was calculated. A doubly robust estimation method was used as sensitivity analysis. Conventionally, both outcome regression and propensity score methods are impartial only if both of the statistical models are accurately defined when applied separately to assess a causal impact. The doubly robust estimator combines the two approaches, and only one of the two models needs to be correctly specified to obtain an unbiased effect estimator (Funk et al., 2011). An inverse probability of treatment weighting (IPTW) cohort was generated using the estimated propensity scores as weights (Chesnaye et al., 2022). Then a logistic regression was performed on the weighted cohort, thus combining a logistic regression model for the propensity of having high RC or low RC with a multivariate regression model for the OR of stroke.

Interaction and subgroup analyses were conducted to evaluate the heterogeneity of the RC effect in subgroups stratified by age (< 65 or ≥65 years) and sex. Subgroup analyses were performed using stratified logistic regression models and interaction across subgroups was tested using the likelihood ratio test. Finally, mediation analyses were performed to evaluate the indirect effect of RC on stroke mediated through hypertension or diabetes, using R package “mediation.”

Overall, R Statistical Software 4.2.1^1^ (The R Foundation) and Free Statistics 1.7 were used for the data analyses described above, and a two-sided value of *p* < 0.05 was considered statistically significant.

## Results

### Baseline characteristics and outcomes

A total of 7,035 participants were finally included in the entire cohort (Table 1). Among them, 3,546 participants exhibited RC levels above the median (i.e., the high RC group). Several clinical characteristics including sex, smoking, diabetes, hypertension, BMI, LDL and hs-CRP differed significantly between the two groups. Individuals in the high RC group were more likely to be male; be a current smoker or drinker; have a higher prevalence of hypertension and diabetes, and have higher BMI. The high RC group exhibited a higher level of hs-CRP and a lower level of LDL. After adjustment with PSM, the baseline covariates were balanced between the two groups (Supplementary Figure S1). After 6 years of follow-up, participants in the high RC group had a higher stroke incidence than the low RC group [in the entire cohort, 51 (1.4%) vs. 27 (0.8%), *p* = 0.007; In the PSM cohort, 40 (1.5%) vs. 21 (0.8%), *p* = 0.014] (Supplementary Figure S2).

**Table 1:** Baseline characteristics of the entire cohort and PSM cohort.

	Entire cohort (*n* = 7,035)				PSM cohort (*n* = 5,280)			
	Low RC (*n* = 3,489)	High RC (*n* = 3,546)	SMD	*P*	Low RC (*n* = 2,640)	High RC (*n* = 2,640)	SMD	*P*
Age, years	50.49 ± 14.45	51.05 ± 13.88	0.040	0.093	50.59 ± 14.04	50.66 ± 14.15	0.005	0.867
Male, *n* (%)	1,506 (43.2)	1764 (49.7)	0.132	<0.001	1,224 (46.4)	1,230 (46.6)	0.005	0.869
Smoking, *n* (%)	897 (25.7)	1,077 (30.4)	0.104	<0.001	742 (28.1)	733 (27.8)	0.008	0.783
Drinking, *n* (%)	1,078 (30.9)	1,236 (34.9)	0.084	<0.001	865 (32.8)	875 (33.1)	0.008	0.770
Diabetes, *n* (%)	225 (6.4)	508 (14.3)	0.260	<0.001	220 (8.3)	214 (8.1)	0.008	0.764
Hypertension, *n* (%)	923 (26.5)	1,211 (34.2)	0.168	<0.001	779 (29.5)	812 (30.8)	0.027	0.322
BMI, kg/m^2^	22.56 ± 3.20	24.23 ± 3.50	0.499	<0.001	23.24 ± 3.17	23.30 ± 3.07	0.018	0.511
LDL, mmol/L	3.08 ± 0.99	2.91 ± 0.96	0.168	<0.001	2.99 ± 0.91	2.99 ± 0.95	0.002	0.951
Hs-CRP, mg/dL	1.0 (0.0, 2.0)	1.0 (1.0, 3.0)	0.117	<0.001	1.0 (0.0, 2.0)	1.0 (1.0, 2.0)	0.012	<0.001

RC, remnant cholesterol; BMI, body mass index; Hs-CRP, high-sensitivity C-reactive protein; LDL, low-density lipoprotein; PSM, propensity score matching; SMD, standardized mean differences. Data were expressed as mean ± SD for continuous variables and numbers (*n*) with percentages (%) for categorical variables. An SMD value < 0.1 was considered balanced.

### Association between RC and stroke

As is shown in Table 2, in the entire cohort, participants in the high RC group had a higher risk of stroke (crude OR, 1.87; 95% CI, 1.17–2.99) compared with those in the low RC group. The association remained significant after adjustment for all covariates in Table 1 (OR, 1.74; 95% CI, 1.06–2.85) and adjustment for propensity score (OR, 1.65; 95% CI, 1.01–2.69). In the PSM cohort, logistic regression showed that high RC was associated with a higher risk of stroke, with an OR of 1.92 (95% CI, 1.13–3.26). The association was consistent using doubly robust estimation method (OR, 1.68; 95% CI, 1.07–2.64).

**Table 2:** Association between RC and stroke.

Analysis	OR (95% CI)	*P*
Entire cohort		
Crude analysis^a^	1.87 (1.17 ~ 2.99)	0.009
Multivariable-adjusted analysis^b^	1.74 (1.06 ~ 2.85)	0.027
Propensity score-adjusted analysis^c^	1.65 (1.01 ~ 2.69)	0.047
**PSM cohort**	1.92 (1.13 ~ 3.26)	0.016
**Doubly robust estimation**	1.68 (1.07 ~ 2.64)	0.025

^a^No variables were adjusted. ^b^Adjusted for variables in Table 1. ^c^Adjusted for propensity score. PSM, propensity score matching.

### Subgroup analyses

**Figure 2:**
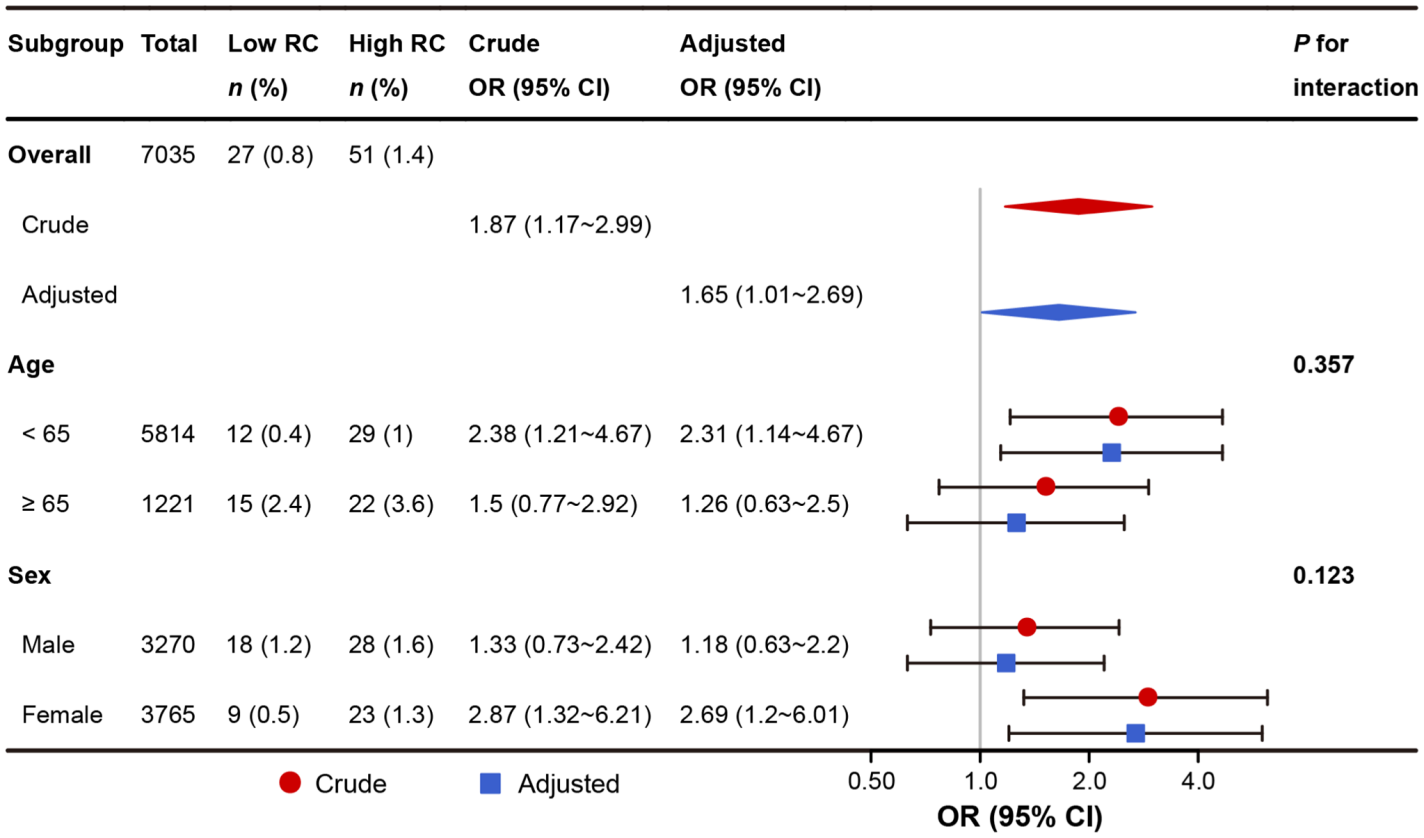
Subgroup analyses of the association between RC and stroke in the entire cohort. RC, remnant cholesterol. In the crude model (red color), no variable was adjusted. In the adjusted model (blue color), the propensity score was adjusted.

The association of RC with stroke was further validated in subgroups stratified by age and sex (Figure 2). No significant interactive effects were observed in the entire cohort before or after adjustment for propensity score (all *p* for interaction >0.05), showing that the effect of RC on stroke was homogeneous across subgroups stratified by age and sex.

### Mediation analyses

**Figure 3:**
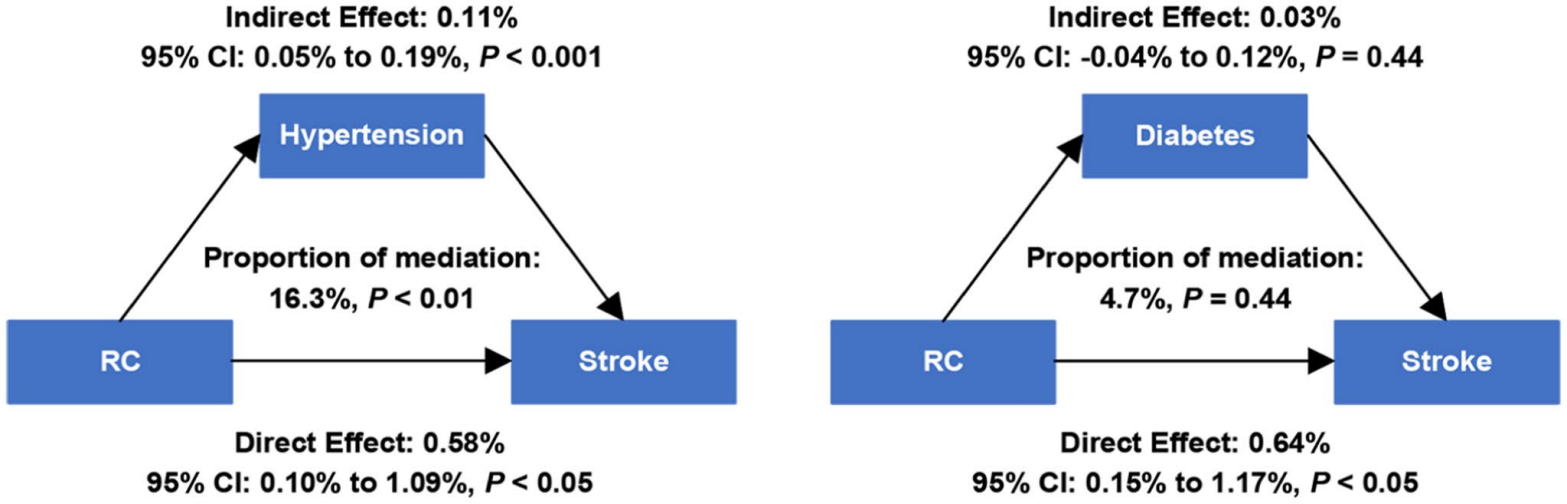
Estimated proportion of the association between RC and stroke mediated by hypertension and diabetes. RC, remnant cholesterol. Models were adjusted for age and sex.

The indirect effect of RC on stroke risk through hypertension or diabetes was detected by mediation analyses (Figure 3). We observed a significant indirect effect of RC on stroke mediated by hypertension (indirect effect = 0.11%; 95% CI, 0.05–0.19%; *p* < 0.001). Data showed hypertension mediated the association of RC with stroke risk, and the proportion of mediation effect was 16.3% (*p* < 0.01). When diabetes was considered as a mediator, no significant indirect effect was observed.

## Discussion

In the present study, we found that high RC levels at baseline were independently associated with an increased risk of first-ever stroke in the Chinese general population. Furthermore, our findings suggest the mediating effect of hypertension might be involved in the association between RC and stroke.

RC refers to the cholesterol content of TRL, which carries both TG and cholesterol. As TG can be degraded by lipoprotein lipase and does not accumulate in the atherosclerotic plaque, it has been argued not casually associated with cardiovascular events (Nordestgaard and Varbo, 2014; Nordestgaard, 2016). RC seems to be a more clinically relevant and promising atherosclerotic predictor. It penetrates vessel walls and becomes trapped under the intima, due to its large size. Cholesterol in RC accumulates in the arteries and is swallowed by macrophages or smooth muscle cells, which leads to foam cell formation (Miller et al., 2010). Pro-atherothrombogenic molecules in endothelial cells, including intercellular adhesion molecule-1, vascular cell adhesion molecule-1, and tissue factor, can be upregulated by RC (Doi et al., 2000). RC induces a procoagulant state by activating platelets and the coagulation cascade (Olufadi and Byrne, 2006). RC can also accelerate endothelial senescence by increasing oxidative stress and induce endothelial dysfunction by inhibiting nitric oxide production (Liu et al., 2009). Moreover, RC promotes the production of pro-inflammatory cytokines and genetic evidence suggests that elevated RC levels are associated with low-grade inflammation (Shin et al., 2004; Varbo et al., 2013). These studies provide mechanistic evidence for confirming the role of RC in atherogenesis.

Numerous studies have found that RC promotes atherosclerotic cardiovascular disease (Castañer et al., 2020; Langsted et al., 2020; Quispe et al., 2021). There were also some observational studies on the relationship between RC and stroke. The Copenhagen General Population Study and the Copenhagen City Heart Study have reported an association between high RC concentration and an increased incidence of ischemic stroke in the Danish population (Varbo and Nordestgaard, 2019). Chen et al. (2021) conducted a cohort study in rural areas of Northeast China and found that participants with higher RC levels had a significantly greater incidence of combined cardiovascular diseases, including fatal and non-fatal stroke and coronary heart disease. In individuals with myocardial infarction or ischemic stroke, lower RC was estimated to reduce recurrent major cardiovascular event incidence rates by 20% in secondary prevention (Langsted et al., 2020). Another study revealed that higher RC variability was associated with a higher risk of ischemic stroke in the general population derived from the employee and retiree populations of the Kailuan Group, a coal mining company in China (Li et al., 2022).

Our findings demonstrated that elevated RC was associated with high first-ever stroke risk in the general population in China, consistent with Chen’s findings (Chen et al., 2021). Rather than employing a regional cohort, data in the present study was derived from a nationwide cohort of CHNS. The CHNS was carried out in 15 provinces and municipal cities across China varying substantially in geography, economic development, public resources, and health indicators. Therefore, the results of the present study might be generalized to the Chinese population with caution. Furthermore, we used propensity score-based methods and doubly robust estimation method to reduce bias.

Numerous studies have revealed that increased RC is generally associated with hypertension and diabetes. The levels of RC are significantly increased in patients with diabetes or hypertension (Schaefer et al., 2002; Xu et al., 2021). Some recent data have shown that high RC was associated with higher blood pressure, as well as a higher risk of diabetes (Kasahara et al., 2013; Li et al., 2021; Xie et al., 2021; Hu et al., 2022; Szili-Torok et al., 2022). A recent cross-lagged analysis has indicated that increased RC might precede the development of hypertension (Chen et al., 2022). In our study, we observed that the population with high RC was more likely to be accompanied by a high incidence of diabetes or hypertension. To evaluate whether RC contributes to stroke *via* hypertension or diabetes, mediation analyses were conducted. It is interesting to note that hypertension might be a mediator between RC and stroke, and the estimated proportion of mediating effect was 16.3%. The mechanism of RC-induced hypertension is not fully clarified, potentially involving vascular damage, oxidative stress, inflammation, insulin resistance and the renin-angiotensin system (Chen et al., 2022), while the role of hypertension in stroke has been well recognized. Therefore, we speculate that hypertension caused by elevated RC might be partially involved in the onset and development of stroke, adding a possible mechanism for the association between RC and stroke.

There are still some limitations in the present study. Firstly, the causal role of RC in stroke risk cannot be ascertained, because of its observational design. Secondly, self-reported stroke occurrence was employed in the present study, and information about stroke subtypes was not available. The contribution of RC to different stroke subtypes needs to be clarified in future research. Thirdly, some participants were excluded for the missing outcome status due to the limitation of available data from CHNS. The accurate time of the outcome event could not be determined, which limited the application of Cox proportional hazard models in the analyses. Fourthly, residual confounding that has not been measured may still exist in this study even if propensity score-based methods were used. Moreover, the study population was exclusively Chinese and the results might not be generalizable to other populations.

## Conclusion

Our study concludes that increased RC is independently associated with first-ever stroke in the Chinese general population. Notably, this effect might be partially mediated by hypertension. These findings highlight the importance of recognizing high RC and provide a rationale for controlling RC in the primary prevention of stroke. Further studies are required to examine the trajectory of RC and its association with stroke.

## Data availability statement

The datasets generated and/or analyzed during the current study are available in the CHNS database repository: https://www.cpc.unc.edu/projects/china.

## Ethics statement

The studies involving human participants were reviewed and approved by the institutional review boards of the University of North Carolina at Chapel Hill and the National Institute of Nutrition and Food Safety, and the Chinese Center for Disease Control and Prevention. The patients/participants provided their written informed consent to participate in this study.

## Author contributions

R-SD: study conception and design. HL, SM, LC, BL, and Y-BL: acquisition, analysis, or interpretation of data. HL and SM: statistical analysis. HL: manuscript drafting. Y-BL and R-SD: review and comment to manuscript. All authors read and approved the final manuscript.

## Funding

This work was supported by Academic promotion programme of Shandong First Medical University (2019QL013).

## Conflict of interest

The authors declare that the research was conducted in the absence of any commercial or financial relationships that could be construed as a potential conflict of interest.

## Publisher’s note

All claims expressed in this article are solely those of the authors and do not necessarily represent those of their affiliated organizations, or those of the publisher, the editors and the reviewers. Any product that may be evaluated in this article, or claim that may be made by its manufacturer, is not guaranteed or endorsed by the publisher.

## Footnotes

^1^ http://www.R-project.org
