# Supplementary material for: Association and mediating mechanism between remnant cholesterol and first-ever stroke among the Chinese general population
Source: Front Neurosci. 2023 May 25;17:1161367. doi: 10.3389/fnins.2023.1161367 (PMC10247974; doi:10.3389/fnins.2023.1161367)
Supplement: Supplementary file 1 [file Data_Sheet_1.docx]

Supplementary Material

Association and mediating mechanism between remnant cholesterol and first-ever stroke among the Chinese general population

Heng Li , Shuai Miao, Lu Chen; Bin Liu; Yan-Bin Li; Rui-Sheng Duan^*^

*** Correspondence:** Rui-Sheng Duan: ruisheng_duan@163.com


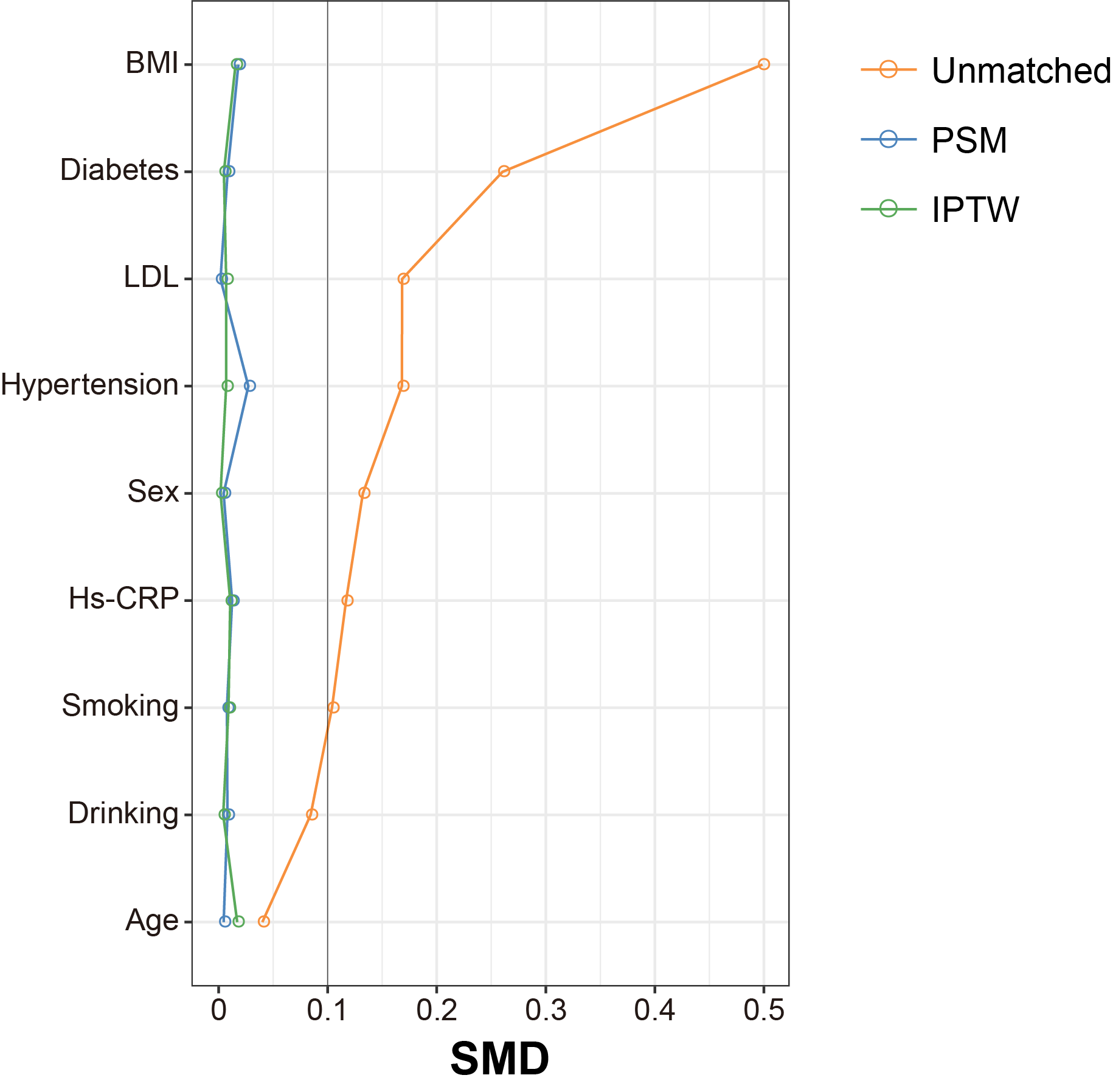


**Supplementary Figure 1.** Distribution of matching factors between the low RC and high RC groups.

RC, remnant cholesterol; BMI, body mass index; LDL, low-density lipoprotein; Hs-CRP, high-sensitivity C-reactive protein; PSM, propensity score matching; IPTW, inverse probability of treatment weighting. Group differences were assessed using standardized mean differences (SMD), and an SMD value < 0.1 was considered balanced.

**
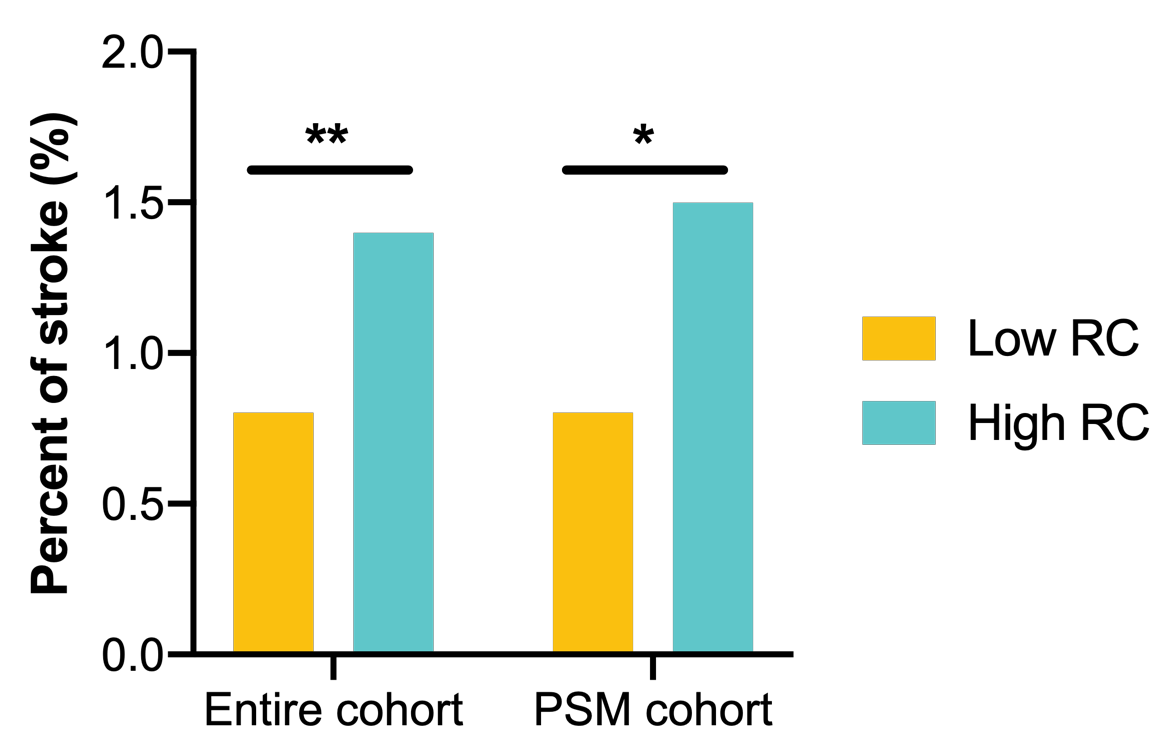
**

**Supplementary Figure 2.** Percent of participants with stroke in high RC and low RC groups.

RC, remnant cholesterol; PSM, propensity score matching. (**p* < 0.05, ***p* <0.01)
